# Climate modelling and structural stability

**DOI:** 10.1007/s13194-021-00414-0

**Published:** 2021-10-19

**Authors:** Vincent Lam

**Affiliations:** 1 Institute of Philosophy & Oeschger Centre for Climate Change Research, University of Bern, CH-3012 Bern, Switzerland; 2 School of Historical and Philosophical Inquiry, The University of Queensland, QLD 4072, St Lucia, Australia

**Keywords:** Climate models, Structural stability, Structural model error, Hawkmoth effect, Topology, Dynamical systems theory, Chaos theory, Climate projections, Decision-making

## Abstract

Climate modelling plays a crucial role for understanding and addressing the climate challenge, in terms of both mitigation and adaptation. It is therefore of central importance to understand to what extent climate models are adequate for relevant purposes, such as providing certain kinds of climate change projections in view of decision-making. In this perspective, the issue of the stability of climate models under small relevant perturbations in their structure (or small relevant ‘structural model errors’ with respect to the target system) seems particularly important. Within this framework, a debate has emerged in the philosophy of science literature about the relevance for climate modelling of the mathematical notion of structural stability. This paper adresses several important foundational and epistemological questions that arise in this context, in particular about the the role of abstract mathematical considerations of a qualitative nature (in some precise, topological sense) for concrete modelling projects with mainly quantitative purposes.

## Introduction

Climate modelling plays a crucial role for understanding and addressing the climate challenge, in terms of both mitigation and adaptation. It is therefore of central importance to understand to what extent climate models are adequate for relevant purposes (Parker, 2009), such as providing certain kinds of climate change projections in view of decision-making. In this perspective, the issue of the stability of climate models under small relevant perturbations in their structure (or small relevant ‘structural model errors’ with respect to the target system) seems particularly important. Besides difficulties for the decision making relevance of certain climate change projections, issues related to structural stability (and structural model error) may also point, in certain cases, towards some kind of epistemically relevant fundamental (irreducible) limitations of climate modelling (McWilliams, 2007). Such ‘in-principle’ limitations (with respect to certain purposes) would not be easily mitigated by making climate models more comprehensive and more complex (e.g. by including more processes) or by increasing computational power.

These issues are known to the climate science community, where they seem however to be rather little discussed (see Smith 2002 for an example)—they actually often fall in the general set of issues labelled ‘structural (model) uncertainty’. Part of the reasons may come from the fact that the rather abstract and involved mathematical framework of dynamical systems theory underlying structural stability issues is quite far removed from the concerns of the working climate scientists and climate modellers (indeed, these questions tend to be confined to the sub-community interested in the mathematical foundations of climate science and climate modelling, see e.g. Ghil et al. (2008) and Majda et al. (2010)) and also from the fact that the concrete impact of these issues can be rather difficult to quantify.

Interestingly, a debate has emerged in the philosophy of science literature about the relevance (in particular for climate modelling) of structural model error and structural stability. Indeed, beyond the difficult purely technical (mathematical) issues, there is also a number of very interesting more conceptual questions.

On the one hand, Frigg et al. (2014) construct a thought experiment or toy example using the logistic map in order to illustrate the critical impact of structural model error on (probabilistic) model outputs (and on their relevance for decision making).^1^ Their argument for the general scope of the critical impact of structural model error beyond the kind of thought experiment they put forward crucially relies on considerations about (the absence of) structural stability (Frigg et al., 2014, §4)—in this context, the critical impact on the decision-making relevance of the combination of structural model error and lack of structural stability is often broadly (and sometimes a bit loosely) called ‘hawkmoth effect’ in the literature, in reference but also in distinction to the famous ‘butterfly effect’.^2^ Relatedly, issues about structural model error and structural stability lie in the background of the critical assessment of the UKCP09 climate modelling project in Frigg et al. (2013b) and Frigg et al. (2015).

On the other hand, Winsberg and Goodwin (2016), Goodwin and Winsberg (2016), and Nabergall et al. (2019) raise a number of issues and object to the general relevance of the hawkmoth effect for climate projections; in particular, they challenge the general scope (in view of decision-making) of the absence of (a proof of) structural stability.^3^ This paper does not aim to settle all aspects of this debate—somewhat surprisingly, beyond the bold claims, it seems that both sides actually agree that structural model error can be a real worry in certain cases, one of the main points of disagreement being rather about the scope of the worry and the precise role of structural stability.

This paper aims to highlight the epistemic relevance of structural stability considerations within the framework of climate modelling. To this aim, after introducing the notions of structural model error and structural stability, we will discuss their role in the hawkmoth effect argument, with a particular attention to the crucial (but little discussed) notion of ‘genericity’ in this context (Section 2). We will then address a series of issues related structural stability that have been raised within the framework of the debate about the hawkmoth effect, and we will argue that these issues do not affect the general epistemic relevance of investigating structural stability features of climate models (Section 3). We will put the discussion in a broader perspective in Section 4.

Before starting, a note on our focus on climate modelling: the issues discussed here are of course not specific to climate modelling, but the latter provides an interesting study case, not least because of the important potential implications in the climate change context, e.g. in terms of what to expect from climate modelling in view of decision making and addressing the climate challenge.

## Structural stability and the hawkmoth effect

Climate models obviously differ in many ways from a perfect representation of the target climate system (because of, e.g., discretization, approximations, missing physical processes, …).^4^ We are here interested in certain structural differences. In particular, a model has ‘structural model error’ (SME) if its “functional form is relevantly different from that of the true system” (Frigg et al., 2014, 35).^5^ The dynamics of a model with SME differs from the dynamics of the true (target) system in a way that “can destroy the utility of [the] model’s predictions” (Frigg et al., 2014, 39): as already mentioned in the previous section, when combined with considerations linked to structural stability, this is often broadly referred to as the ‘hawkmoth effect’ (mainly in the philosophy of science literature), in reference but also in contrast to the well-known butterfly effect. The intuition underlying this reference (and contrast) is that the hawkmoth effect is about sensitive dependence on model structure, whereas the butterfly effect is about sensitive dependence on initial conditions (the notion of ‘sensitive dependence’ needs to be precisely articulated in both cases).^6^Frigg et al. (2014) further argue that the hawkmoth effect cannot be easily mitigated with probabilistic methods, in contrast to the butterfly effect where, very roughly, the sensitive dependence to initial conditions can be mitigated by considering (the evolution of) a probability distribution over initial conditions.

The argument for the general relevance of the hawkmoth effect beyond specific cases (and in particular for climate modelling) crucially relies on mathematical considerations from dynamical systems theory involving the notion of structural stability (Frigg et al., 2014, §4). Indeed, dynamical systems theory allows the study of the qualitative behaviour of physical (mechanical) systems, in particular the study of the geometrical and topological properties of their phase portrait (the set of all trajectories of a dynamical system over time in state space). In this context, dynamical systems theorists (and their precursors) have developed the notion of structural stability in order to address qualitative issues of the following kind:

If a dynamical system *X* has a known phase portrait *P*, and is then perturbed to a slightly different system *X*^′^ (for example, changing the coefficients in its differential equation slightly), then is the new phase portrait *P*^′^ close to *P* in some topological sense? This problem is of obvious importance, since in practice the qualitative information obtained for *P* is to be applied not to *X*, but to some nearby system $X^{\prime }$, because the coefficients of the equation may be determined experimentally or by an approximate model and therefore approximately. (Abraham and Marsden, 1978, xix)

Suppose our dynamical system is the solution of a differential equation or otherwise comes from a real world physical system. Ordinarily, the system itself will be only a model of real world phenomena: certain assumptions will have to be made, and certain approximations and experimental errors will be present. Hence the dynamical system itself, albeit a completely accurate solution of the physical model, will nevertheless be only an approximation to reality since the model itself suffers this flaw. Now, if the dynamical system in question is not structurally stable, then the small errors and approximations made in the model have a chance of dramatically changing the structure of the real solution to the system. That is, our “solution”could be radically wrong or unstable. (Devaney, 1989, 53)

Intuitively, the above mentioned worry related to SME in climate modelling can be rephrased along similar lines within the framework of dynamical systems theory: is the phase portrait of a climate model (understood as a dynamical system) with (small) SME close in some topological sense to the phase portrait of the dynamical system we are ultimately interested in, namely the target climate system? Or, in other terms: is the dynamics described by the considered climate model with (small) SME qualitatively equivalent (‘close in some topological sense’) to the dynamics of the target climate system? The notion of structural stability precisely provides a way to characterize when this is the case.^7^

If on the other hand, the dynamical system in question is structurally stable, the small errors introduced by approximations and experimental errors may not matter at all: the solution of the model system may be equivalent or topologically conjugate to the actual solution. (Devaney, 1989, 53)

In intuitive terms, a dynamical system is structurally stable if its dynamics remains qualitatively equivalent under small perturbations (of course, ‘qualitatively equivalent’ and ‘small’ need to be made precise in this context). Let us now consider a somewhat more formal characterization of structural stability. We first need to define the relevant equivalence relation (we here closely follow Katok & Hasselblatt, 1995, 68-69).

### **Definition 1**

For *r* ≥ 0 two *C*^*r*^ maps $f: M \rightarrow M$ and $g: M \rightarrow M$ are said to be *topologically conjugate* if there exists a homeomorphism $h: M \rightarrow M$ such that *f* = *h*^− 1^ ∘ *g* ∘ *h*.^8^

*M* typically is a smooth manifold, which can represent the state (phase) space of the considered dynamical system, *f* and *g* can represent possible time evolutions for this system (e.g., in our context, the model dynamics with SME and the ‘true’ one of the target system).^9^ One can similarly define topological conjugacy for flows on phase space (the homeomorphism has to map orbits of one flow onto orbits of the other while preserving the orientation).^10^ The important point to highlight here is that if two dynamical system evolutions are topologically conjugate, then they have the same (asymptotic) dynamical properties, such as the same dynamical invariants (e.g. same fixed points with same properties). We can now define the structural stability condition.

### **Definition 2**

A *C*^*r*^ map *f* is *C*^*m*^ *structurally stable* (1 ≤ *m* ≤ *r*) if there exists a neighbourhood *U* of *f* in the *C*^*m*^ topology such that every map *g* ∈ *U* is topologically conjugate to *f*.

Again, structural stability can be similarly defined for flows on phase space.^11^ We can now clearly see the relevance of structural stability for the general scope of the hawkmoth effect. Indeed, if the model dynamics is not structurally stable, then even if it has small SME (with respect to the relevant topology) and so is topologically close to the dynamics of the target system, its dynamics can be topologically inequivalent to the one of the target system. As a consequence, the model can then have a radically different dynamical behaviour from the one of the target system and therefore can be totally inadequate for providing decision relevant projections.^12^

A crucial step in the argument based on structural stability for the general relevance of the hawkmoth effect relies on mathematical results suggesting that structural stability is not generic among dynamical systems in dimensions greater than 2. In this context, one can say that a property is generic in a set *S* if for any element *p* ∈ *S* the property is either satisfied or is satisfied for some element in any open neighbourhood of *p* (in intuitive terms, the property is either satisfied for *p* or for an element that is ‘arbitrarily close’ in some topological sense).^13^ The claim that structurally stable dynamical systems are not generic is in particular motivated by a theorem due to Smale, which basically shows that there exists an open set *U* of dynamical systems that are not structurally stable (Smale, 1966):^14^ so any element of *U* is a counterexample for the genericity of structural stability among dynamical systems. It follows directly from this result that “there are some dynamical systems that cannot be approximated by structurally stable ones” (Pugh & Peixoto, 2008).

Within the framework of climate modelling, these considerations therefore suggest, the argument goes, that structural stability is not generic (in the precise sense defined above) among climate models (understood as dynamical systems) and that the target climate system itself could not be approximated (in the topological sense) by structurally stable models.^15^ Because of complexity and dimensionality issues (among others), it is however extremely difficult to provide rigorous stability results in the climate context.^16^ But still, the mathematical considerations discussed above can be considered as strong indications that nice stability properties should not be assumed to be generic (in the precise topological sense discussed here) in the climate context.

As a consequence, intuitively, one could be tempted to say that ‘most’ dynamical systems (in dimensions greater than 2) are therefore not structurally stable. Great care is required, though. ‘Most’ is generally understood in measure-theoretic terms, but such measure-theoretic characterization raises subtle questions in the present setting since the ‘space of dynamical systems’ is infinite-dimensional. In this context, it is therefore more appropriate to introduce a notion of genericity that is topological rather than measure-theoretic, as we do here. Specifically, Smale’s theorem mentioned above is an ‘openness’ and ‘density’ result rather than a measure-theoretic one: in particular, it does imply that one can be sure that the property of ‘structural stability’ is not generic in the set of all dynamical systems, so that there are for sure dynamical systems that cannot be approximated by structurally stable ones. Having said that, Smale’s theorem does not prevent having certain classes of climate models that are indeed structurally stable, because it does not rule out the existence of classes of structurally stable systems that are open and dense in an appropriate (proper) subset of all systems (of non-zero measure in some appropriate measure). The extent to which these considerations may impact our epistemic attitude with respect to certain climate model outputs and their decision-making relevance is in many ways at the heart of the matter (see Section 3).^17^

We can now summarize the three main steps (HE_1_)–(HE_3_) in the hawkmoth effect argument, which allow to highlight the role of structural stability considerations.

- Climate models have structural model error (SME).
- Structural stability is not generic among dynamical systems; since climate models can be considered as dynamical systems, one should not expect them to exhibit structural stability in general.^18^
- Given SME, lack of structural stability can lead to certain climate model predictions/projections being misleading and irrelevant for decision-making.

Strictly speaking, ‘lack of structural stability’ should be understood as ‘lack of appropriate stability features’, as we will discuss in the next section. Also, note that (HE_2_) can be replaced by: (HE2′) Structural stability is not generic among dynamical systems; since the target climate system can be considered as a dynamical system, one should not expect the climate system to exhibit appropriate stability features in general.

The first step (HE_1_) is uncontroversial. Several issues related to structural stability have been raised in the literature in connection to (HE_2_) and mainly (HE_3_)—we address these issues in the next section.

## The epistemic relevance of stability features in climate modelling

In the debate around the hawkmoth effect, a series of issues have been raised in relation to structural stability.^19^ These issues point to fundamental aspects of structural stability as well as to important open questions in climate modelling. The aim of this section is to discuss these issues focusing on structural stability and to argue that they do not affect the general epistemic relevance of appropriate stability features within climate modelling. We consider three main (sets of) issues.

- *No relevant analogy between lack of structural stability and chaos.* The first issue concerns the analogy between the butterfly effect and the hawkmoth effect: assuming that the relevance of the latter for climate projections relies on being analogous to the former, disputing this analogy amounts to disputing the relevance of the hawkmoth effect itself. To this aim, the strategy in Nabergall et al. (2019, §3) is to consider standard features of chaotic systems underlying the butterfly effect and then to stress the difficulties to identify similar features in the case of the hawkmoth effect. The focus lies in particular on relevant degrees of sensitive dependence to initial conditions and on topological mixing; for instance, it is pointed out that there is no clear counterpart to the notion of exponential sensitive dependence in the case of lack of structural stability.^20^ There is no need to enter into the subtle technical details of chaos theory here; the interesting point for us is that the lack of structural stability involves a priori no notion allowing to evaluate how ‘big’ and how ‘fast’ the discrepancies can grow.^21^ The worry then is that the related deficiencies in predictive capacities cannot be quantified, and so are irrelevant for climate projections.
- *Structural stability is about topological rather than metrical features.* The second issue is closely related to the first: indeed, according to (1), lack of structural stability is disanalogous to chaos because the first concerns topological features and topological features are just not the right kind of features that are relevant for predictions. The fact that structural stability is about topological features just follows from its definition in terms of topological conjugacy (see definition 2 in Section 2). Of course, a failure of topological conjugacy as such involves no metrical information. So, the argument goes, a model’s time evolution that fails to be topologically conjugate to the time evolution of the considered target system may still be predictively reliable; lack of structural stability, as a topological rather than metrical notion, is therefore of little or of no relevance for predictions/projections.

Regarding the first issue (1), two points are worth making here. First, it should be noted that chaos can well be characterized topologically, so that the topological nature alone of structural stability (and lack thereof) cannot be what grounds any potential disanalogy between lack of structural stability and chaos. Second and most importantly, a qualitative difference may be relevant (e.g. for climate projections and decision-making) even if it does not exhibit features analogous to chaotic ones.^22^

More generally, there is a common difficulty with the two issues (1)–(2) as (somewhat schematically) exposed above, which revolves around the implicit background assumption that a topological difference (in the sense of lack of topological conjugacy) has in general no relevant metrical implication in this context.^23^ But, clearly, this may not always be the case. Indeed, the topological character of the structural stability property implies that a model that is not structurally stable may well display some qualitatively different behaviour from the target system (e.g. with a different attractor structure) in a way that renders the model’s predictions/projections unreliable (e.g. in view of decision-making)—note that, at this stage, introducing a metric in order to ‘quantify’ things may not be needed in order to draw relevant (qualitative) conclusions about the reliability of certain models projections (for certain purposes).

Now, a crucial question includes the prevalence of such unreliability induced by the lack of structural stability. For instance, given a concrete climate model with SME, what are the exact implications of its (generic)^24^ lack of structural stability features on the reliability of its projections? It is in general extremely difficult to answer precisely (rigorously) to such questions—for reasons partly related to issues discussed under (3) below. But this difficulty does not affect the above considerations, that is, the general epistemic relevance of appropriate stability features (or lack thereof) for certain climate modelling purposes (see also the discussion below).

- *Issues about the class of models, the notion of similarity, and the time scale.* Structural stability is a feature that is relative to the class of models considered, as well as to the choice of topology (and to the choice of the metric, if applicable) for defining a notion of similarity (and for defining a metrical notion of closeness, if applicable). For instance, it follows directly from our definition of structural stability in Section 2 that it is dependent on the choice of topology (see definition 2); in intuitive terms, the latter defines what can be regarded as a small perturbation or a small SME.^25^ Analogously, the choice of the class of models is crucial to the definition of structural stability. These considerations then raise the following issue: either the worry about the lack of structural stability is merely an artefact of considering a class of models that is too broad^26^ or the question of the appropriate class is just too indeterminate to reach any meaningful conclusion. On top of this issue, there is the further question of the time scales at which the lack of structural stability may become relevant for predictive (and related decision-making) purposes. Again, the nature of the worry is similar:^27^ either the time scales at which the lack of structural stability would have an impact is not relevant for the predictive purposes we are interested in (e.g. climate change projections for certain variables and certain emission scenarios at the the end of the 21^st^ century) or we simply have no clue; in both cases, it may seem that the epistemic relevance of structural stability considerations is much weakened.

This set of issues points to difficult and still open questions linked to the exact implications of the lack of structural stability for concrete climate projections (and related decision-making). However, the fact that these issues remain open actually calls for further investigations about the manifestation of appropriate stability features (and lack thereof) in concrete climate models. For instance, concerning the relevant time scales, a possible first step can be to investigate how the various sources of uncertainties (for certain variables) are partitioned for different lead times (e.g., Hawkins and Sutton 2009 argue that, for decadal mean surface temperature,^28^ model uncertainty tend to dominate over natural internal variability for longer lead times); such investigations may provide certain indications about the time scales at which issues linked to SME and the lack of structural stability may manifest themselves. In the end, however, much depends on the specifics of the particular climate modelling situation under consideration.^29^

Globally, the discussion of the issues (1)–(3) above tends to show that questions related to structural stability—and, more generally, qualitative features of the climate models phase portrait—do require careful attention in the climate modelling context, in particular in view of climate decision-making. However, it should be clear that highlighting the (possible) implications of the lack of structural stability constitutes in no way an argument for imposing structural stability as a necessary condition for a climate model to be adequate for certain predictive tasks (this would possibly disqualify most of them).^30^ The considerations above (as well as the discussion in Section 2) rather motivates ensuring that the model’s concrete predictions or projections are *sufficiently* stable (for a certain purpose) and are not the result of some ‘structurally unstable’ behaviour (in some sense to be made precise)—thereby highlighting the epistemic relevance of appropriate stability features in climate modelling.^31^

These considerations are reminiscent of the critical discussions in dynamical systems theory in the last century around the so-called ‘stability dogma’, according to which “structurally unstable systems were regarded as somehow suspect” so that “structural stability was *imposed* as an *a priori* restriction on “good” models of physical phenomena” (Guckenheimer & Holmes, 1983, 259; see also Abraham & Marsden, 1978, xix-xx).^32^ But as such this dogma seems too strict (especially given the lack of genericity of structural stability in dimension greater than two), even if its underlying epistemic motivation is sound. This latter can be preserved in a weakened version, for instance following Guckenheimer and Holmes (1983, 259), who suggest to reformulate the stability dogma “to state that the only properties of a dynamical system (or a family of dynamical systems) which are *physically relevant* are those which are preserved under perturbations of the system”.^33^

Similarly, in the philosophy of science literature, Fletcher (2020) recently defends a principle of stability as an epistemological principle “partially constitutive of the activity of representational modeling itself” (16). It follows from Fletcher’s principle that appropriate stability considerations are crucial for justifying a model’s predictions; this is well in line with the upshot of the discussion above. Moreover, Fletcher’s topological formalization of the principle allows to explicitly highlight the importance for stability issues of the topology and the class of models that are considered—which correspond, as we have seen above, to some fundamental questions in climate modelling.

## Perspectives

We would like to conclude with two important perspectives, at two different levels, that are strengthened by taking seriously the question of structural stability in the climate modelling context. At the mathematical and foundational level, structural stability considerations have further motivated a stochastic perspective on climate modelling. In this approach, a stochastic version of structural stability is considered, which takes into account random perturbations, for instance related to unresolved processes or linked to natural or anthropogenic forcing; the underlying expectation is that some level of added noise can improve (the understanding of) the (stochastic) stability features of the climate models and their projections (see Ghil et al., 2008 for instance; this approach is also well in line with the stochastic perspective on parameterization, see recently Berner et al., 2017 and Palmer, 2019 as well as references therein). This stochastic approach to stability issues is being developed within the general framework of random dynamical systems theory (Arnold, 1998), which typically allows to describe (nonautonomous) dynamical systems with stochastic forcing; more generally, the stochastic (and statistical) perspective in climate science and climate modelling actually highlights the relevance of mathematical investigations in this context.^34^

At the epistemic and decision-making level, issues related to the lack of structural stability further highlight the importance of a qualitative perspective on climate models, especially in view of reliable climate projections and appropriate decision-making. To the extent that they point to some fundamental epistemic limitations of climate modelling (McWilliams, 2007), uncertainties linked to SME and lack of structural stability further stress the crucial role of understanding—or “process understanding” (Knutti, 2018)—and background knowledge in building confidence in climate projections (Baumberger et al., 2017). Relatedly, these uncertainties and limitations also strengthen the case for systematically including expert knowledge in providing relevant model based support for decision-making in the climate context (Thompson et al., 2016, Thompson and Smith 2019; how precisely to incorporate expert judgement in a systematic way raises several open questions though). It is important to stress that, in many ways, these qualitative perspectives can be considered as a crucial aspect of climate modelling itself—they of course imply in no way that modelling is useless.

So, issues related to structural stability (and lack thereof) in climate science and climate modelling raise many important foundational and epistemological questions that require careful investigations. A preliminary list of such questions includes the following points: spelling out the extent to which stability features are required for a certain purpose and in a certain context, exploring the kind of stability (and lack thereof) involved in concrete modelling projects (as well as the characteristic time scales at play)^35^, and further developing the qualitative (in the mathematical sense) study of climate models. With respect to this last point, it should be highlighted that stability issues—and more generally the qualitative approach in the sense of dynamical systems theory, and bifurcation theory in particular—are also central to the understanding of abrupt climate changes and tipping points.^36^ In view of the climate challenge, these questions need to be taken seriously.

## Footnotes

1 See also Frigg et al. (2013a) and Bradley et al. (2014); the very relevance of this thought experiment is debated in the philosophy of science literature.

2 The expression ‘hawkmoth effect’ has been introduced in Thompson (2013). We make the hawkmoth effect argument explicite in Section 2.

3 See also the paragraph 5.6 as well as the appendix in Winsberg (2018).

4 Of course, a climate model does not aim to perfectly represent the target climate system, but only the aspects that are relevant for the specific purposes the model has been designed for (see Parker 2009; see also the discussion from a climate scientist’s point of view in Knutti (2018)).

5 Structural model error is about model structure and so is not equivalent to parameter uncertainty.

6 The famous expression ‘butterfly effect’ is generally associated with Edward Lorenz, who may have actually meant something rather different from ‘mere’ sensitive dependence to initial conditions, see Palmer et al. (2014).

7 There are many different notions of stability in dynamical systems theory, which rely on different equivalence relations (more on that below). In the fascinating development of dynamical systems theory, these notions primarily aim at classifying dynamical systems with respect to these equivalence relations (and at characterizing their orbit structure), see the standard references (Abraham & Marsden, 1978, ch. 7) and (Katok & Hasselblatt, 1995, ch. 2). Besides this purely mathematical perspective, there is also clearly a practical and epistemic motivation to the investigations of stability properties in dynamical systems, as exemplified by the quotes in the main text (see also, e.g., Robbin 1972, [§1]); about this epistemic motivation, see Fletcher (2020).

8 A map $h: A \rightarrow B$ (where *A*,*B* are topological spaces or smooth manifolds) is a *homeomorphism* if it is continuous, bijective and its inverse is also continuous; in intuitive terms, a homeomorphism between two spaces captures the idea that they are similar from the topological point of view. A map is of differentiability class *C*^*r*^ if its derivatives up to the order *r* exist and are continuous.

9 Katok & Hasselblatt (1995, 68) consider *f* and *g* on different manifolds *M* and *N* respectively; this changes nothing for our discussion.

10 In general, flows describe continuous time dynamics whereas maps—and iterations thereof—describe discrete time dynamics.

11 Indeed, Pugh and Peixoto (2008) proposes the following general “Bourbaki-style” definition: “If a set is equipped with a topology and an equivalence relation then its **structurally stable** elements are those interior to the equivalence classes. The “structure” is whatever is preserved by the equivalence relation; its structure remains the same when a structurally stable element is perturbed.” In the case of flows, as mentioned above in the text, the relevant equivalence relation is orbit preserving.

12 The precise nature of the modal aspect here is epistemologically crucial in many ways. The discussion about genericity which follows lies at the heart of the issue.

13 In an alternative measure-theoretic perspective, one can say that a property is generic in a set *S* if the ensemble in which it is not satisfied is of zero measure. However, as we will discuss below, the topological characterization of ‘genericity’ proposed in the main text is more appropriate for the context here.

14 “MAIN THEOREM. There exists a compact 4 dimensional manifold *M*, an open set *U* in the space of *C*^*r*^ vector fields, *C*^*r*^ topology, *r* > 0, on *M* such that no *X* ∈ *U* is structurally stable.” (Smale, 1966, 491) As already mentioned above, dynamical systems can be represented by flows and their associated vector fields.

15 As we will discuss in this paper, this implies in no way that climate models are useless.

16 For an explicite expression of this difficulty, see McWilliams (2007, 8711) for instance: “Although we may expect a chaotic AOS [atmospheric and oceanic simulation] model to be structurally unstable, it is difficult to explicitly make this determination. The attractor cannot be fully visualized or measured because the phase space has such a high dimension (i.e., high order).”

17 I am grateful to an anonymous referee for pushing me on this.

18 Thanks to an anonymous referee for highlighting the structure of this second step to me.

19 See Winsberg and Goodwin (2016), Goodwin and Winsberg (2016), and Nabergall et al. (2019), as well as the paragraph 5.6 and the appendix in Winsberg (2018).

20 See Werndl (2009) for a discussion of chaos theory in the philosophy of science literature: in intuitive terms, sensitive dependence to initial conditions “means that small errors in initial conditions lead to totally different solutions” (203), and topological mixing “means that any bundle of solutions spread out in phase space like a drop of ink in a glass of water” (204).

21 Such an evaluation requires a metric, which allows to measure distances, e.g. in phase space; no metric is required to discuss structural stability.

22 Mayo-Wilson (2015) also discusses the disanalogies between lack of structural stability and various notions of chaos; he makes clear that this leaves open the question of the “importance of the various notions of structural stability for prediction, control, and explanation” (1244).

23 The issues (1)–(2) seem to rely on a sort of dichotomy between topological and metrical aspects, but this need not be the case: the two are actually best conceived as complementary.

24 See the discussion about the topological notion of ‘genericity’ in Section 2.

25 In the recent philosophy of science literature—albeit in a slightly different context—Fletcher (2020, §4) clearly highlights the dependence of stability considerations on the choice of topology.

26 Discussing structural stability as defined in Section 2 (see definition 2), Katok and Hasselblatt (1995, 69) are very explicit: “Attempts to either replace topological conjugacy by smooth equivalence or to allow arbitrary continuous maps or even arbitrary homeomorphism as perturbations lead to vacuous notions.”

27 It should be clear however that the issues are rather different, e.g. the issue of the relevant time scales being a more practical question for instance.

28 The partitioning of uncertainty depends on the variable of interest, but also on the region and the spatial scale.

29 In this sense critical assessment of particular climate modelling projects, such as in Frigg et al. (2013b) and Frigg et al. (2015), is extremely valuable; however, in general, these issues are not very often investigated in great depth in concrete cases.

30 So, strictly speaking, the challenge posed by Frigg et al. (2014, 47) that “those using non-linear models for predictive purposes have to argue that the model they use is one that is structurally stable” seems hopeless; an alternative understanding of Frigg et al. (2014) here can possibly be articulated in terms of appropriate stability features with respect to some predictive context, very much along the lines of what is argued for here.

31 See Fletcher (2020) for a recent discussion of the epistemic motivations for stability features (encoded in a ‘principle of stability’) in a general modelling context.

32 In his famous book *The Aim and Structure of Physical Theory* first published in French in 1908, Duhem argues for a criterion of stability for models “to be useful to the physicist” (1991, 143); if Schmidt (2011) explicitly understands Duhem’s criterion of stability as a precursor to the stability dogma (see also Abraham & Marsden, 1978, xix-xx), Fletcher (2020, §3.3) argues for a more epistemological reading, following which Duhem’s criterion is about what can be inferred from the models rather than about what is possible according to the models.

33 See also the discussion in Batterman (2002, §4.4).

34 Methods relying on the mathematical (and statistical physics) framework of the fluctuation-dissipation theorem have also been exploited in this context; for instance, Majda et al. (2010) develop a linear statistical response approach based on the fluctuation-dissipation theorem explicitly in order to deal with the lack of structural stability in climate modelling—showing *en passant* that this latter is seriously investigated by some researchers working on the foundations of climate science and climate modelling.

35 As already discussed in relation to (3) in Section 3, this is a central question, since lack of structural stability may become ‘manifest’ (e.g. for decision-making purposes) only asymptotically or at time scales that are irrelevant for the concrete modelling and decision-making purposes under consideration in a given context.

36 E.g. see Ashwin et al. (2012), Bathiany et al. (2016) and recently Ghil and Lucarini (2020), in particular the parts III & V.

## References

[CR1] Abraham, R., & Marsden, J.E. (1978). *Foundations of Mechanics* (2nd edn). Addison-Wesley Publishing Company.

[CR2] Arnold, L. (1998). *Random dynamical systems*. Springer.

[CR3] Ashwin P, Wieczorek S, Vitolo R, Cox P (2012). Tipping points in open systems: Bifurcation, noise-induced and rate-dependent examples in the climate science. Philosophical Transactions of the Royal Society A.

[CR4] Bathiany, S., Dijkstra, H.A., Crucifix, M., Dakos, V., Brovkin, V., Williamson, M.S., Lenton, T.M., & Scheffer, M. (2016). Beyond bifurcation: Using complex models to understand and predict abrupt climate change. *Dynamics and Statistics of the Climate System*, *1*, dzw004.

[CR5] Batterman, R.W. (2002). *The devil in the details: Asymptotic reasoning in explanation, reduction and emergence*. Oxford University Press.

[CR6] Baumberger C, Knutti R, Hirsch Hadorn G (2017). Buidling confidence in climate model projections: An analysis of inferences from fit. WIREs Climate Change.

[CR7] Berner (2017). Stochastic parameterization: Towards a new view of weather and climate models. Bulletin of the American Meteorological Society.

[CR8] Bradley, S., Frigg, R., & Du, H. (2014). Model Error and Ensemble Forecasting: A Cautionary Tale. In G.C. Guo C. Liu (Eds.) *Scientific Explanation and Methodology of Science* (pp. 58–66). World Scientific.

[CR9] Devaney, R.L. (1989). *An Introduction to Chaotic Dynamical Systems* (2nd edn). Addison-Wesley Publishing Company.

[CR10] Duhem, P. (1991). *The aim and structure of physical theory*. Princeton University Press.

[CR11] Fletcher S (2020). The principle of stability. Philosophers’ Imprint.

[CR12] Frigg R, Bradley S, Du H, Smith LA (2014). Laplace’s demon and the adventures of his apprentices. Philosophy of Science.

[CR13] Frigg, R., Bradley, S., Machette, R.L., & Smith, L.A. (2013a). Probabilistic forecasting: Why model imperfection is a poison pill. In H. Anderson, D. Dieks, G. Wheeler, W. Gonzales, & T. Uebel (Eds.), *New Challenges to Philosophy of Science* (pp. 479–491). Springer.

[CR14] Frigg R, Smith LA, Stainforth DA (2013). The myopia of imperfect climate models: The case of UKCP09. Philosophy of Science.

[CR15] Frigg R, Smith LA, Stainforth DA (2015). An assessment of the foundational assumptions in high-resolution climate projections: The case of UKCP09. Synthese.

[CR16] Ghil M, Chekroun MD, Simonnet E (2008). Climate dynamics and fluid mechanics: Natural variability and related uncertainties. Physica D.

[CR17] Ghil M, Lucarini V (2020). The physics of climate variability and climate change. Reviews of Modern Physics.

[CR18] Goodwin WM, Winsberg E (2016). Missing the forest for the fish: How much does the ‘hawkmoth effect’ threaten the viability of climate projections?. Philosophy of Science.

[CR19] Guckenheimer, J., & Holmes, P. (1983). *Nonlinear oscillations, dynamical systems and bifurcations of vector fields*. Springer.

[CR20] Hawkins E, Sutton RT (2009). The potential to narrow uncertainty in regional climate predictions. Bulletin of the American Meteorological Society.

[CR21] Katok, K., & Hasselblatt, B. (1995). *Introduction to the Modern Theory of Dynamical Systems*. Cambridge University Press.

[CR22] Knutti, R. (2018). Climate model confirmation: From philosophy to predicting climate in the real world. In E. A. Lloyd, & E.Winsberg (Eds.), *Climate modelling: Philosophical and conceptual issues* (pp. 325–359). Cham.

[CR23] Majda AJ, Abramov R, Gershgorin B (2010). High skill in low-frequency climate response through fluctuation dissipation theorems despite structural instability. Proceedings of the National Academy of Sciences.

[CR24] Mayo-Wilson C (2015). Structural chaos. Philosophy of Science.

[CR25] McWilliams J (2007). Irreducibe imprecision in atmospheric and oceanic simualtions. Proceedings of the National Academy of Sciences.

[CR26] Nabergall, L., Navas, A., & Winsberg, E. (2019). An antidote for hawkmoths: On the prevalence of structural chaos in non-linear modeling. *European Journal for Philosophy of Science* 9.

[CR27] Palmer TN (2019). Stochastic weather and climate models. Nature Reviews Physics.

[CR28] Palmer TN, Döring A, Seregin G (2014). The real butterfly effect. Nonlinearity.

[CR29] Parker W (2009). II–Confirmation and Adequacy-for-Purpose in Climate Modelling. Aristotelian Society Supplementary Volume.

[CR30] Pugh C, Peixoto M (2008). Structural stability. Scholarpedia.

[CR31] Robbin JW (1972). Topologcal conjugacy and structural stability for discrete dynamical systems. Bulletin of the American Mathematical Society.

[CR32] Schmidt, J.C. (2011). Challenged by Instability and Complexity. In C. Hooker (Ed.), *Philosophy of Complex Systems*. Oxford.

[CR33] Smale S (1966). Structurally stable systems are not dense. American Journal of Mathematics.

[CR34] Smith LA (2002). What might we learn from climate forcasts?. Proceedings of the National Academy of Sciences.

[CR35] Thompson, E.L. (2013). *Modelling north atlantic storms in a changing climate* [PhD thesis]. Imperial College.

[CR36] Thompson EL, Frigg R, Helgeson C (2016). Expert judgment for climate change adaptation. Philosophy of Science.

[CR37] Thompson EL, Smith LA (2019). Escape from model-land. Economics: The open-access. Open Assessment E-Journal.

[CR38] Werndl C (2009). What are the new implications of chaos for unpredictability. British Journal for the Philosophy of Science.

[CR39] Winsberg, E. (2018). *Philosophy and climate science*. Cambridge University Press.

[CR40] Winsberg E, Goodwin WM (2016). The adventures of climate science in the sweet land of idle arguments. Studies in History and Philosophy of Modern Physics.

